# Plasma concentrations of soluble cytokine receptors in euthymic bipolar patients with and without subsyndromal symptoms

**DOI:** 10.1186/1471-244X-12-158

**Published:** 2012-09-26

**Authors:** Turan Cetin, Sinan Guloksuz, Esin Aktas Cetin, Sema Bilgic Gazioglu, Gunnur Deniz, E Timucin Oral, Jim van Os

**Affiliations:** 1 Research, Treatment and Training Center for Alcohol and Substance Dependence (AMATEM), Bakirkoy Research and Training Hospital for Psychiatry, Neurology and Neurosurgery, Istanbul, Turkey; 2 Department of Psychiatry and Psychology, Maastricht University Medical Centre, EURON, Maastricht, The Netherlands; 3 Department of Immunology, Institute of Experimental Medicine (DETAE), Istanbul University, Istanbul, Turkey; 4 Department of Psychology, Faculty of Arts and Sciences, Istanbul Commerce University, Istanbul, Turkey; 5 King’s College London, King’s Health Partners, Department of Psychosis Studies, Institute of Psychiatry, London, United Kingdom

**Keywords:** Bipolar disorder, Cytokine, Interleukin, Inflammation, Tumor necrosis factor, Euthymic, Subsyndromal, Staging, Biomarker

## Abstract

**Background:**

Current evidence suggests that high concentrations of pro-inflammatory markers are associated with bipolar disorder characterized by severe impairment during inter-episodic periods, reduced treatment response and persistent subsyndromal symptoms. We tested whether persistent subsyndromal symptoms in euthymic bipolar patients were associated with markers of an ongoing chronic pro-inflammatory process.

**Methods:**

Forty-five euthymic bipolar patients (22 with subsyndromal symptoms (BD+) and 23 without subsyndromal symptoms (BD-) and 23 well controls (WC) were recruited for assessment of soluble tumor necrosis factor receptor-1 (sTNF-R1), soluble interleukin-6 receptor (sIL-6R) and soluble interleukin-2 receptor (sIL-2R) concentrations. Soluble cytokine receptor concentrations were assessed using enzyme-linked immunosorbent assay.

**Results:**

In comparison to WC, sTNF-R1 concentration was higher in both BD- and BD+ (age and sex adjusted standardized β, respectively: β = 0.34, p = 0.012 and β = 0.41, p = 0.003). Similarly, compared to WC, sIL-6R concentration was higher in both BD- and BD+ (age and sex adjusted standardized β, respectively: β = 0.44, p = 0.001 and β = 0.37, p = 0.008). There was no difference between BD- and BD+ in the concentration of either sTNF-R1 or sIL-6R; plasma concentration of sIL-2R was not analyzed as 75% percent of the samples were non-detectable.

**Conclusions:**

Although bipolar patients present with a pro-inflammatory shift compared to well controls, subsyndromal symptoms are not associated with additive increasing effects. Longitudinal studies with larger samples are required to clarify the relationship between illness course and inflammatory markers in bipolar disorder.

## Background

Bipolar disorder (BD) has long been considered an episodic illness characterized by complete symptomatic recovery during inter-episodic periods. However, a growing body of evidence shows that the rate of inter-episodic morbidity in the form of subsyndromal symptoms is much higher than previously thought [1,2]. Therefore, early recognition and treatment of subsyndromal symptoms represents an important target for clinicians, given associations with increased risk of relapse, decrement in functioning and cognitive dysfunction [2-4]. However, treatment alternatives are limited for the patients with subsyndromal symptoms, many of whom do not respond adequately to conventional therapies [4].

Recent data indicate that immune modulation may play an important role in the pathophysiology of BD, with the possibility of novel mechanistic options for treatment [5,6]. Alterations in cytokine functioning may represent a fitting theoretical perspective underlying possible immuno-modulatory treatment approaches. Current knowledge about cytokines points to regulatory effects in the central nervous system, similar to their role in the immune system [7]. Studies comparing cytokine concentrations in bipolar patients and healthy controls have demonstrated that mania and depression show characteristics of pro-inflammatory states with higher concentrations of pro-inflammatory cytokines and lower concentrations of anti-inflammatory cytokines [6]. Additionally, up-regulation of the immune system appears to resolve during euthymia [8-11]. In the light of this evidence, inflammation in BD may be regarded as a state arising during acute episodes rather than a trait influencing longitudinal course of illness. However, findings of studies far from consistent; selection of heterogeneous study populations may represent an important factor underlying between-study discrepancies. For example, previous work by our group suggests that concentrations of tumor necrosis factor-alpha (TNF-α), a major pro-inflammatory cytokine, may represent heterogeneity between euthymic BD patients in association with longitudinal measures of lithium response [12]. Indeed, the use of inflammatory markers for staging BD has been proposed [13,14]. According to this model, high concentrations of TNF-α are associated with advanced stages of BD with severe impairment during inter-episodic periods, reduced treatment response and ongoing subsyndromal symptoms.

Therefore, the current study aimed to determine if there is an ongoing chronic pro-inflammatory process in euthymic bipolar patients with subsyndromal symptoms, characterised by higher concentrations of soluble tumor necrosis factor receptor-1 (sTNF-R1), soluble interleukin-6 receptor (sIL-6R) and soluble interleukin-2 receptor (sIL-2R), all of which are thought to represent reliable markers of inflammatory activity and all of which have been widely investigated in BD. Moreover, a recent meta-analysis, examining a wide range of cytokines and soluble cytokine receptors, shows evidence of higher concentrations of sTNF-R1 and sIL-2R in bipolar patients [15]. To this end, the study sampling frame provided comparisons between three groups: (i) euthymic bipolar patients with subsyndromal symptoms (BD+); (ii) euthymic bipolar patients without subsyndromal symptoms (BD-); (iii) well controls (WC).

## Methods

### Study population

A total of 45 patients, who met the DSM-IV criteria for BD-I, were recruited among long-term follow-up outpatients of the Rasit Tahsin Mood Disorders Outpatient Unit (RTMDOU) of the Bakirkoy Research and Training Hospital for Psychiatry, Neurology, and Neurosurgery. All of the 910 registered BD patients at RTMDOU continue to be evaluated with standardized medical forms based on a nation-wide mood disorders follow-up program named SKIP-TURK [16]. The SKIP-TURK form, which is similar to the “Clinical Monitoring Form” (CMF) used in the Systematic Treatment Enhancement Program for Bipolar Disorder (STEP-BD) [17], was put into use to (i) assess the clinical characteristics of BD patients (e.g. polarity of the first episode, duration of illness) and (ii) to evaluate illness course of BD patients over clinical follow-up. The diagnosis of the patients was confirmed both by clinical interview and by the SKIP-TURK procedure. Of the patients, 23 were euthymic without subsyndromal symptoms (BD-). BD+ status was defined *a priori* on the basis of consensus decisions in SKIP-TURK; thus, 22 patients were in a euthymic state with at least 2 moderate affective symptoms coded in the SKIP-TURK form at 2 consecutive clinical evaluations during a follow-up period of minimally two months, however without meeting criteria for a full affective episode as defined in DSM-IV. All the patients were medicated either with mono-therapy of either of mood stabilizer, an antipsychotic or an antidepressant, or with combination therapy combining any of these.

Twenty-three well controls were also recruited from the hospital staff, reflecting the general population socioeconomic strata. A clinical psychiatrist (TC) evaluated well controls with a standard clinical psychiatric interview to screen for psychiatric disorders. The exclusion criterion for well controls was presence of any current Axis I psychiatric disorder. The study was approved by the standing Medical Ethics Committee of Bakirkoy Research and Training Hospital for Psychiatry, Neurology, and Neurosurgery, and carried out in accordance with the Declaration of Helsinki. All the participants gave informed consent before enrolment in the study.

Information including medical history, physical examination, laboratory evaluation including complete blood count, blood chemistry, urinalysis, thyroid function test and electrocardiogram were obtained from all participants in order to evaluate potential exclusion criteria. The exclusion criteria for all participants were any allergic disease, an infectious disease within the last four weeks, use of any potentially immunosuppressive drug such as corticosteroids, non-steroid anti-inflammatory drugs within the last four weeks, pregnancy or breastfeeding, current use of alcohol at more than 5 standard units per week, current intake of caffeine at more than three cups of coffee per day, and current use of tobacco at more than 10 cigarettes a day.

### Enzyme-linked immunosorbent assays (ELISAs) for sTNF-R1 and sIL-6R

The blood samples were obtained between 08.00 AM and 10.00 AM and were collected in heparin vacuum tubes. The blood samples were immediately centrifuged for 10 min at 3000 rpm and the plasma samples were stored at -80°C until analysis. sIL-2R, sIL-6R and sTNF-R1 concentrations in the plasma were assessed using an ELISA kit (BioSource International, Inc, Camarillo, USA) according to the manufacturer’s directions. Plasma samples were diluted 1:100 for sIL-6R and 1:4 for sIL-2R detection in the sample using diluent buffer provided with the ELISA kit. Supplied standards were used to generate the standard curves. The samples and standards were applied to wells. Unbound protein was removed by washing, and biotin-conjugate, followed by horseradish peroxidase-conjugated streptavidin, were added in a step-wise manner. After the color reaction with substrate, the optical density was recorded at 450-nm wavelength with an automated ELISA reader. The absorbance at 450 nm was converted to picograms per millilitre (pg/ml) for sIL-6R, sIL-2R and nanograms per millilitre (ng/ml) for sTNF-R1. The minimal detection limits were: for sIL-2R : 16pg/ml, for sIL-6R : 8 pg/ml and for sTNF-R1 : 0.03 ng/ml.

### Statistical analysis

Plasma concentrations of sIL-6R and sTNF-R1 were detectable in all participants. Plasma concentration of sIL-2R was not analyzed as 75% percent of the samples were non-detectable. Demographic, clinical and treatment characteristics were analyzed by one-way ANOVA, two tailed t-test and chi-square test as indicated. Associations between sTNF-R1 and sIL-6R concentrations and *a priori* selected confounders (sex, age) were assessed using Pearson’s product-moment correlation for age and one-way ANOVA for sex. Pearson’s product-moment correlation was also used to analyze the association between duration of illness and soluble cytokine receptor concentrations. Associations between the three groups (WC, BD-, BD+) and approximately normally distributed sTNF-R1 and sIL-6R concentrations were expressed as standardized regression coefficients (β) from multiple regression procedures with sTNF-R1 and sIL-6R as dependent variable and dummy variables of the three groups as independent variable, the WC group serving as reference. Two-sided statistical significance was set at p < 0.05. STATA version 12.0 (STATA Corporation, College Station, TX, USA) was used to carry out the statistical analyses.

## Results

Table 1 lists the demographic, clinical and treatment characteristics. Compared to BD-, BD+ had a greater number of episodes in general (t(43) = 2.93, p = 0.005), depressive episodes (t(43) = 3.41, p = 0.001) and episodes per year (t(43) = 3.44, p = 0.001). There were no other differences between BD+ and BD- in terms of clinical characteristics.

**Table 1:** Demographic, clinical and treatment characteristics

	**WC**	**BD-**	**BD+**	**P-value**
	**(n = 23)**	**(n = 23)**	**(n = 22)**	
	**Mean (SD) or n (%)**	**Mean (SD) or n (%)**	**Mean (SD) or n (%)**	
**Female sex**	12 (52.2)	13 (56.5)	12 (54.5)	0.957^a^
**Age (years)**	31.65 (5.21)	34.61 (7.28)	36.86 (7.03)	0.034^b^
**Age at onset (years)**		22.13 (6.37)	21.64 (6.21)	0.793^c^
**Onset episode (mania/depression)**		15 (65.2) / 8 (34.8)	11 (50) / 11 (50)	0.302^a^
**Duration of illness (years)**		12.48 (7.23)	15.23 (7.65)	0.222^c^
**Total number of episodes**		4.69 (3.08)	8.95 (6.21)	0.005^c^
** Mania**		2.96 (2.14)	4.09(4.62)	0.293^c^
** Depression**		1.13 (1.79)	3.45 (2.70)	0.001^c^
** Mixed**		0.09 (0.29)	0.45 (1.22)	0.168^c^
**Total number of episodes per year**		0.35 (0.17)	0.55 (0.23)	0.001^c^
**Family history of mood disorder**		10 (43.5)	10 (45.5)	0.894^a^
**Treatment modality**				0.006^a^
** MS**		9 (39.13)	1 (4.55)	
** MS + AP**		12 (52.17)	13 (59.09)	
** MS + AD**		2 (8.70)	2 (9.09)	
** MS + AP + AD**		0	6 (27.27)	
**Duration of ongoing subsyndromal symptoms (months)**			4.95 (3.43)	
**Subtype of ongoing subsyndromal symptoms (depressive/manic/mixed)**			14/6/2	

*WC*: well controls, *BD*-: euthymic bipolar patients without subsyndromal symptoms, *BD*+: euthymic bipolar patients with subsyndromal symptoms, *MS*: Mood stabilizer, *AP*: Antipsychotic, *AD*: Antidepressant.

^a^Chi square.

^b^One-way ANOVA.

^c^t test.

**Figure 1:**
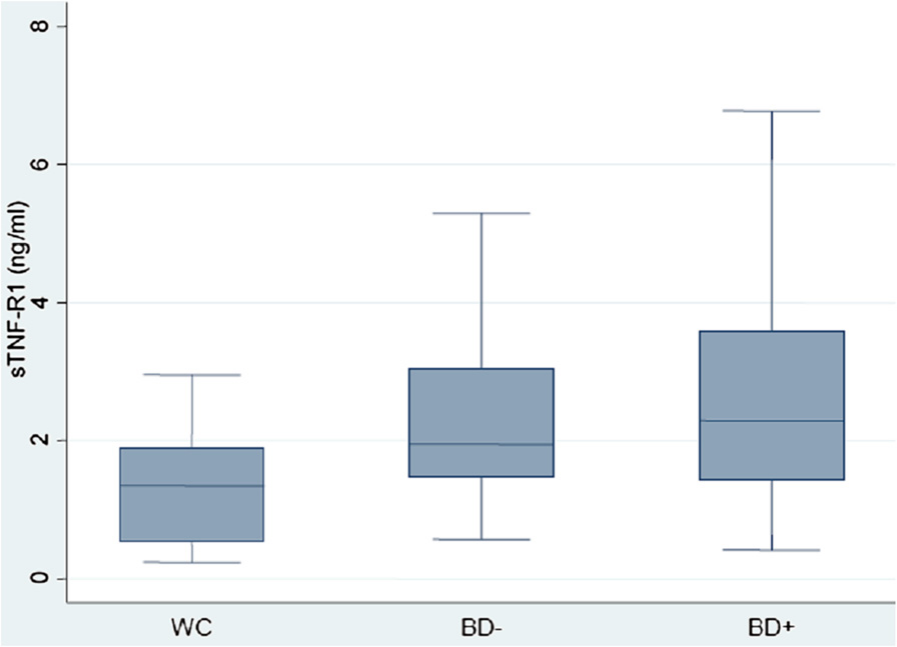
**Box-plot of plasma sTNF-R1 concentration in WC, BD- and BD+.** Median levels are indicated by horizontal lines. WC: well controls, BD-: euthymic bipolar patients without subsyndromal symptoms, BD+: euthymic bipolar patients with subsyndromal symptoms.

**Figure 2:**
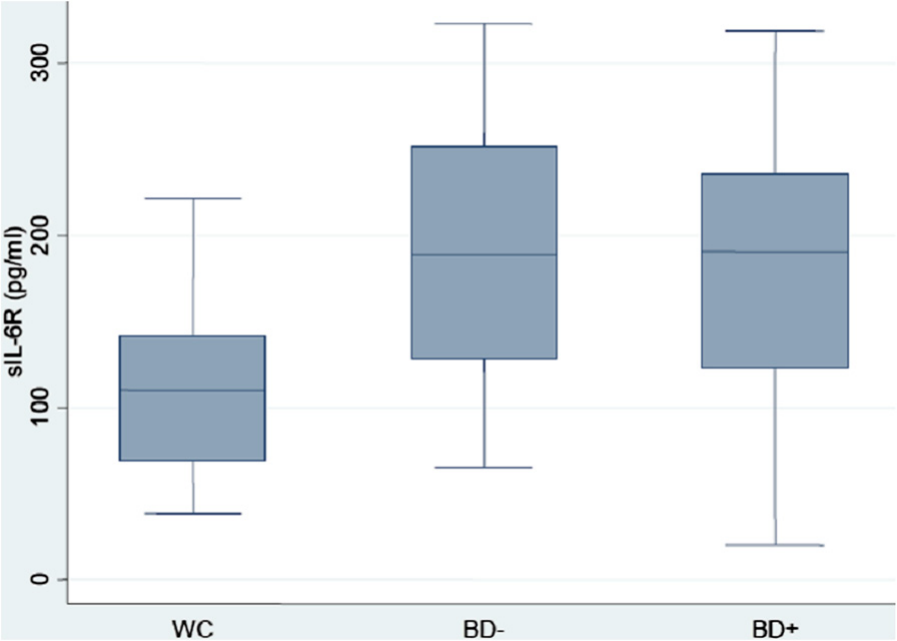
**Box-plot of plasma sIL-6R concentration in WC, BD- and BD+.** Median levels are indicated by horizontal lines. WC: well controls, BD-: euthymic bipolar patients without subsyndromal symptoms, BD+: euthymic bipolar patients with subsyndromal symptoms.

Age was associated with both sTNF-R1 concentration (*r* = 0.30, p = 0.013) and sIL-6R concentration (*r* = 0.25, p = 0.038). There was no strong or significant association between sex and either sTNF-R1 concentration (*F*(1,66) = 0.04, p = 0.835) or sIL-6R concentration (*F*(1,66) = 0.02, p = 0.877). The mean sTNF-R1 concentration was 1.26 (SD = 0.74), 2.31 (SD = 1.13) and 2.62 (SD = 1.64) ng/ml in WC, BD- and BD+, respectively (Figure 1). The mean sIL-6R concentration was 116.12 (SD = 48.46), 192.04 (SD = 72.69) and 184.78 (SD = 82.62) pg/ml in WC, BD- and BD+, respectively (Figure 2). In comparison to WC, sTNF-R1 concentration was higher in BD- and BD+ (uncontrolled standardized β, respectively: β = 0.37, p = 0.005 and β = 0.48, p < 0.001; age and sex adjusted standardized β, respectively: β = 0.34, p = 0.012 and β = 0.41, p = 0.003). There was no difference between BD- and BD+ in sTNF-R1 concentration (uncontrolled standardized β: β = 0.10, p = 0.403; age and sex adjusted standardized β: β = 0.07, p = 0.559). In comparison to WC, sIL-6R concentration was higher in BD- and BD+ (uncontrolled standardized β, respectively: β = 0.47, p < 0.001 and β = 0.42, p = 0.001; age and sex adjusted standardized β, respectively: β = 0.44, p = 0.001 and β = 0.37, p = 0.008). There was no difference between BD- and BD+ in sIL-6R concentration (uncontrolled standardized β: β = -0.05, p = 0.726; age and sex adjusted standardized β: β = -0.08, p = 0.587). There was no strong or significant association between duration of illness and either sTNF-R1 concentration (*r* = 0.20, p = 0.183) or sIL-6R concentration (*r* = 0.08, p = 0.585).

## Discussion

This study demonstrated that there were no differences between BD- and BD+ in terms of sTNF-R1 and sIL-6R concentrations. However, sTNF-R1 and sIL-6R concentrations were higher in both BD- and BD+ in comparison to WC.

Our findings are in line with a previous study reporting higher concentrations of sTNF-R1 in euthymic BD patients compared to controls [18,19]. It has been suggested that soluble cytokine receptors may better reflect the activity of the cytokines in question, given that they are detectable in plasma even when cytokine concentrations are undetectable [18]. Thus, it is plausible to think of soluble cytokine receptors as more reliable markers of activity. In the light of this evidence, both sTNF-R1 and sIL-6R can be considered as markers reflecting pro-inflammatory activity. Both TNF-α and IL-6 are primary regulators of pro-inflammatory process due to their initiating roles in the inflammatory cascade of the acute phase response [7]. They have the ability of affecting various cells and therefore are regarded as possessing pleiotropic properties. It has been demonstrated that the degradation of tryptophan (TRP) by the indoleamine 2,3-dioxygenase (IDO) enzyme is mainly induced by interferon gamma (IFN-γ), but also, to a degree, by TNF-α and IL-6 [20]. Induced IDO activity causes degradation of TRP, which serves as a precursor for serotonin. Moreover, induced IDO activity leads to the formation of neurotoxic metabolites, 3-hydroxykynurenine and quinolinic acid [21]. Several studies have shown an association between IDO activation and mood disorder, particularly unipolar depression [20]. Recently, the TNF-α system has been postulated as a target for understanding the pathophysiology of BD [6,22]. TNF-α exerts its effects via the cell surface receptors called TNFR1 and TNFR2. The effect of TNFR2 is negligible to TNFR1, since TNFR1 is expressed in most cells, whereas TNFR2 is expressed only in hematopoietic cells. TNFR1 mediates apoptosis, cytokine production and activation of nuclear factor kappa B (NF-κB), which induces transcription of cell survival genes (i.e. cellular inhibitors of apoptosis (cIAP), B-cell lymphoma 2 (Bcl-2) family) [6]. It has been postulated that there is a shift towards apoptosis during mood episodes and that this shift arises from increased TNF-α concentration and cessation of NF-κB neuronal translocation due to decreased brain-derived neurotrophic factor (BDNF) and nerve growth factor (NGF) [22]. On balance, the TNF-α system may be considered an important factor playing a role in the regulation of neuroplasticity. Moreover, the notion of TNF-α as a trait marker of BD has some validity given evidence of higher TNF-α concentration in patients with BD than in healthy controls, evidence of effects of TNF-α antagonists on mood symptoms and effects of psychotropics on TNF-α [5,6]. However, it is still premature to count TNF-α as a trait marker of BD due to the influence of several confounding factors, particularly the use of medication, on the immune system. Likewise, the results of our study should be interpreted carefully considering medication use as a potential confounder [23]. Controlling for this was not possible, as only patients used medication, resulting in collinearity. Recent studies showing no difference between medicated euthymic BD patients and well controls in terms of TNF-α and IL-6 concentrations contradict the current findings [8,10,11,24]. The conclusion is that additional studies evaluating inflammatory markers in the euthymic state, ideally comparing medication-free BD patients and matched controls in large samples, are still needed in order to clarify if ongoing inflammation is a trait in BD.

According to the model for staging BD with the use of biomarkers, progression through later stages with social and cognitive dysfunction and persistent subsyndromal symptoms in the inter-episode period is associated with increased oxidative stress and inflammation, which can be demonstrated by high 3-nitrotyrosine and TNF-α concentrations [13,14]. This model stages BD in five categories according to clinical features: Latent Stage is defined by positive family history of mood disorders, subthreshold mood or anxiety symptoms; Stage 1 is defined by euthymic periods without overt psychiatric symptoms; Stage 2 is defined by symptoms during inter-episodic periods and comorbid psychiatric disorders; Stage 3 is defined by marked impairment in cognition and functioning; Stage 4 is defined by need of a caregiver in daily life due to severe impairment in cognition and functioning [13]. The patients with subsyndromal symptoms in our study had persistent symptoms during euthymia, and experienced a greater number of episodes. Thus, they can be considered as patients presenting at a later stage of illness than the group of patients without subsyndromal symptoms. However, we did not find any differences between these two groups in terms of sTNF-R1 and sIL-6R concentrations. In their evaluation of brain-derived neurotrophic factor and cytokines in early and late stage BD patients, Kauer-Sant’Anna and colleagues demonstrated that TNF-α concentration remained higher in late stage patients even after controlling for subsyndromal symptoms [25]. Consequently, they propose that higher TNF-α concentration in the later stages of illness may be due to the cumulative effect of past mood episodes or the cumulative effect of pro-inflammatory shifts throughout the course of illness, rather than the effect of subsyndromal symptoms. In accordance with this finding, the current analysis confirms that subsyndromal symptoms have minimal effect on cytokine concentrations. Nevertheless, we do not underestimate the cumulative effect of ongoing subsyndromal symptoms in patients with advanced duration of illness. BD+ patients had a greater number of episodes per year, which can be counted as an identifier for poor prognosis in the future. Thus, it is also plausible to think that pro-inflammatory shifts may become more evident later in the course of illness in BD+ compared to BD-. Alternatively, BD- and BD+ could belong to same stage, in contrast to what was hypothesized. An in-depth evaluation of social and cognitive functioning could have provided more variables for empirical staging.

To the best of our knowledge, this is the first study investigating immune changes in subsyndromal BD patients. Although our analyses focused on potential inflammatory markers of subsyndromal symptoms in BD-I, no actual associations were presented between inflammatory markers and specific symptoms. Instead, we relied on group-level comparisons of patients with and without subsyndromal symptoms. To the degree that these groups differ in other aspects other than symptomatology, results could have been confounded. Therefore, follow-up research focusing on comparisons at the symptom level in larger samples is required. Although the study had the benefit of recruiting patients with subsyndromal symptoms with the help of standardized assessments in a specialized mood disorders outpatient unit, the definition for subsyndromal state recommended by the International Society of Bipolar Disorder (ISBD) could have been measured more precisely [26]. However, there was no consensus on the definition of subsyndromal states when the study recruitment was initiated. Moreover, the cross-sectional design of the study limits our further prediction about the effect of subsyndromal symptoms on cytokines in the long term. It is also important to emphasize that we were unable to assess body mass index as a potential confounder. Only soluble receptors of TNF-α and IL-6 were assessed, which are the most studied cytokines in BD and considered as molecular targets [6,15,22]. Nevertheless, evaluation of additional inflammatory markers could provide the opportunity of interpreting the findings in more detail. All patients were on medication. As psychotropics (i.e. lithium) may impact on cytokine concentrations [9,12,23], this could have confounded the results. Given the fact that almost all patients in this small study had unique, non-overlapping, treatment modalities (i.e. different combinations of medications and/or different doses), it was impossible to group according to treatment modality in detail and describe medication-related effects in the patient sample. Moreover, previous work suggests that medications have different, sometimes interacting effects on immune mediators [23]. Although it could be argued that medication equivalents might have been used in analyses within the patient group, this method can only be applied to antipsychotics. Furthermore, caution must be taken when using this methodology, since medication equivalents reflect efficacy rather than biological mechanisms. Thus, it was decided not to analyze the effect of each separate medication on immune mediators in this relatively small sample. This is a limitation. On the other hand, eliminating this possibly confounding effect is nearly impossible given the fact that augmentation strategies, resulting in patient-unique medication mixes, are routine practice in the treatment of bipolar disorder [15]. The cross-sectional design of the study also prevented us to evaluate the effects of earlier, currently discontinued treatment. Given the possibility of residual confounding by medication, and the difficulties in disentangling confounding by medication within patient-specific polypharmacy approaches, follow-up semi-experimental studies are required to settle this issue. Finally, the sample size of the study was relatively small, which had the inherent risk of type-II error.

## Conclusions

Despite its preliminary character, findings of our study appear to indicate that subsyndromal symptoms do not influence sTNF-R1 and sIL-6R concentrations. Nevertheless, longitudinal studies with larger samples are required in order to clarify the relationship between illness course and inflammatory markers in BD.

## Competing interests

All authors declare that they have no potential conflict of interest.

## Authors’ contributions

TC and ETO designed the study and wrote the protocol. EAC, SBG and GD commented on the protocol. TC undertook clinical evaluations. EAC and SBG carried out the immunoassays. SG and JVO undertook the statistical analysis. SG wrote the first draft of the manuscript. ETO and JVO commented on the manuscript. All authors contributed to and have approved the final manuscript.

## Pre-publication history

The pre-publication history for this paper can be accessed here:

http://www.biomedcentral.com/1471-244X/12/158/prepub
